# Minimally invasive esophagectomy with non-invasive ventilation by laryngeal mask-assisted anesthesia for esophageal squamous cell carcinoma: case report

**DOI:** 10.3389/fonc.2024.1344662

**Published:** 2024-05-10

**Authors:** Weibi Che, Jian Zhong, Jiawei Huang, Huilong Chen, Caihou Feng, Yujie Xie, Haiquan He, Ying Chen, Cui Li, Bomeng Wu, Wei Ding, Wanli Lin

**Affiliations:** ^1^ Department of Thoracic Surgery, Gaozhou People’s Hospital, Guangdong, China; ^2^ Department of Anesthesiology, Gaozhou People’s Hospital, Guangdong, China

**Keywords:** esophageal squamous cell carcinoma, non-intubation, spontaneous ventilation, minimally invasive esophagectomy, case report

## Abstract

Minimally invasive esophagectomy for cancer surgery remains associated with significant morbidity and surgical complications across the globe. Non-intubation video-assisted thoracic surgery (NIVATS) has been successfully employed in lung resection in recent years, but there are few reported cases with regard to the safety and feasibility of this approach in radical esophagectomy for patients with esophageal cancers. We present 4 consecutive cases with esophageal squamous cell carcinoma (ESCC) who received minimally invasive McKeown’s esophagectomy under non-intubation general anesthesia from November 2022 to April 2023. All these patients were aged from 55 to 75 years old and were pathologically diagnosed with ESCC. All procedures of McKeown’s esophagectomy in these patients were completed with non-invasive ventilation by laryngeal mask-assisted anesthesia. Operation duration ranged from 185 to 395 minutes and the estimated blood loss ranged from 25 to 60 ml in these 4 cases. No severe hypoxia was observed and transient hypercapnia was resolved intraoperatively. None of them was converted to endotracheal intubation with mechanical ventilation or to thoracotomy. The number of retrieved lymph nodes in mediastinum were 21-27 and all patients received R0 surgery with pathological stage as T1bN0M0 to T3N2M0. There was no serious complication (Clavien-Dindo grade III-IV) observed perioperatively and they were all discharged 11-14 days after the surgery with resumption of oral feeding. They are all alive without tumor recurrence at the date of data collection. The safety and efficacy of minimally invasive esophagectomy with non-invasive ventilation by laryngeal mask-assisted anesthesia for patients with ESCC are warranted for explored in a larger cohort study.

## 1 Introduction

Although esophagectomy is the cornerstone of treatment for patients with esophageal cancers, it is notorious for its high invasiveness and high mortality rate (1). Esophagectomy with double-lumen endotracheal intubation and one lung mechanical ventilation is the preferred approach in esophageal surgery at most cancer center (2). Great efforts have been made, for instance, total minimally invasive esophagectomy and robotic-assisted esophagectomy, to minimize the influence of radical esophagectomy on the physical function in patients with esophageal cancers in recent years (3). But the complication rate remains 41%-48%, with major complication (Clavien-Dindo classification ≥ III) ranged from 10%-12% (4), revealing that to shorten the recovery time after esophagectomy and reduce the incidence of postoperative complication still challenge the anesthetist and surgeons. Nonintubated video-assisted thoracoscopic surgery recently has been demonstrated to be a safe and feasible approach for various thoracic diseases (5, 6), but the application of this procedure in esophageal surgery has not been well depicted.

## 2 Case presentation

We report a case series who received radical esophagectomy with non-intubation general anesthesia and discontinuous spontaneous ventilation. These four patients were admitted with progressive dysphagia. After histopathological diagnosis with esophageal squamous cell carcinoma (ESCC) by gastroscopy, these patients routinely received preoperative examinations including enhanced contrast CT, cardiovascular ultrasound, pulmonary function and so on to rule out contraindication for radical esophagectomy. These patients were diagnosed with locally advanced ESCC (cT1b-3N1-2M0) and refused neoadjuvant therapy. Minimally invasive esophagectomy and two field lymphadenectomy under non-incubated anesthesia with discontinuous spontaneous ventilation was performed after acquisition of informed consents. This anesthesia procedure was approved by the Institutional Review Board of our hospital.

**Figure 1:**
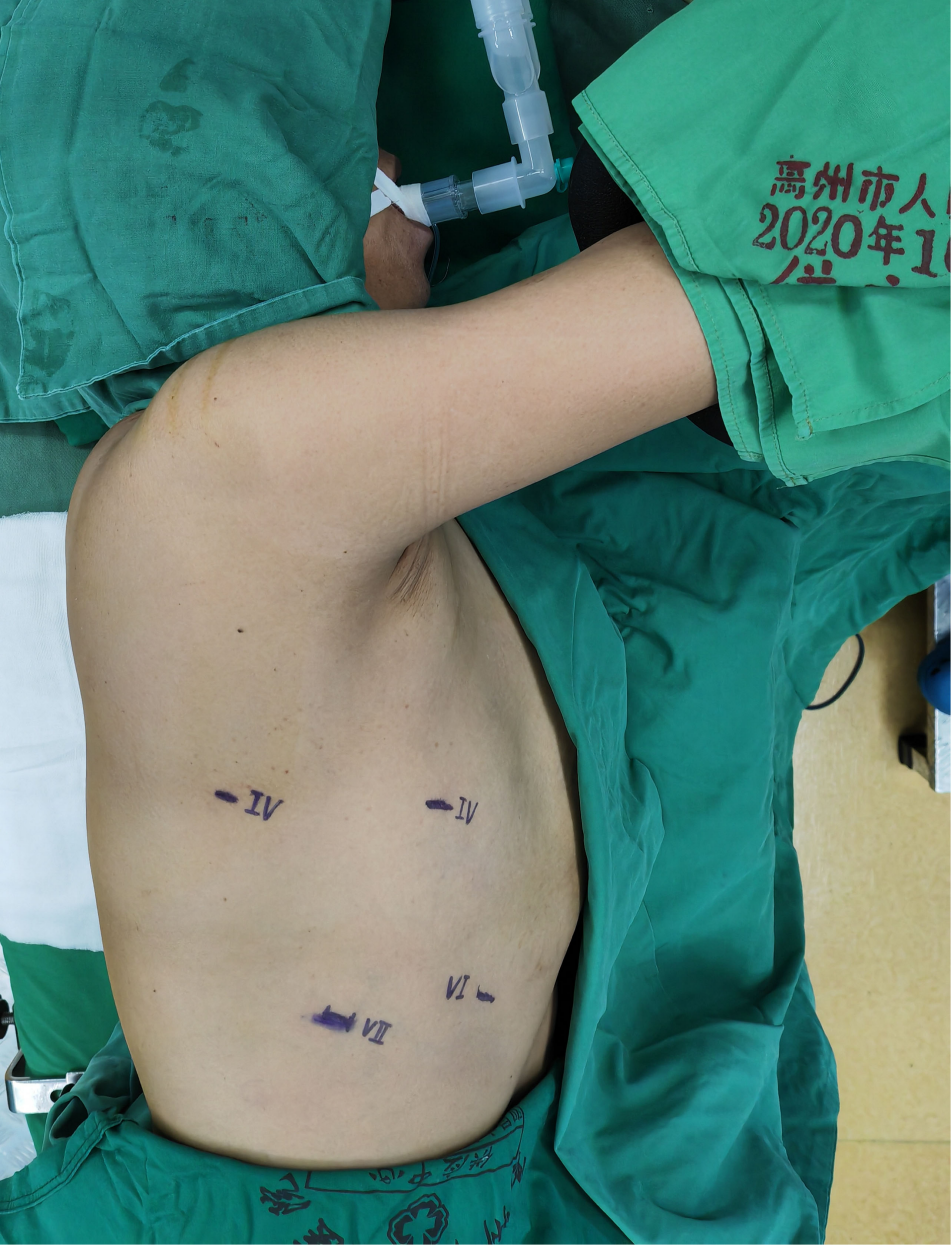
Non-intubation general anesthesia with spontaneous ventilation was performed by laryngeal mask.

**Figure 2:**
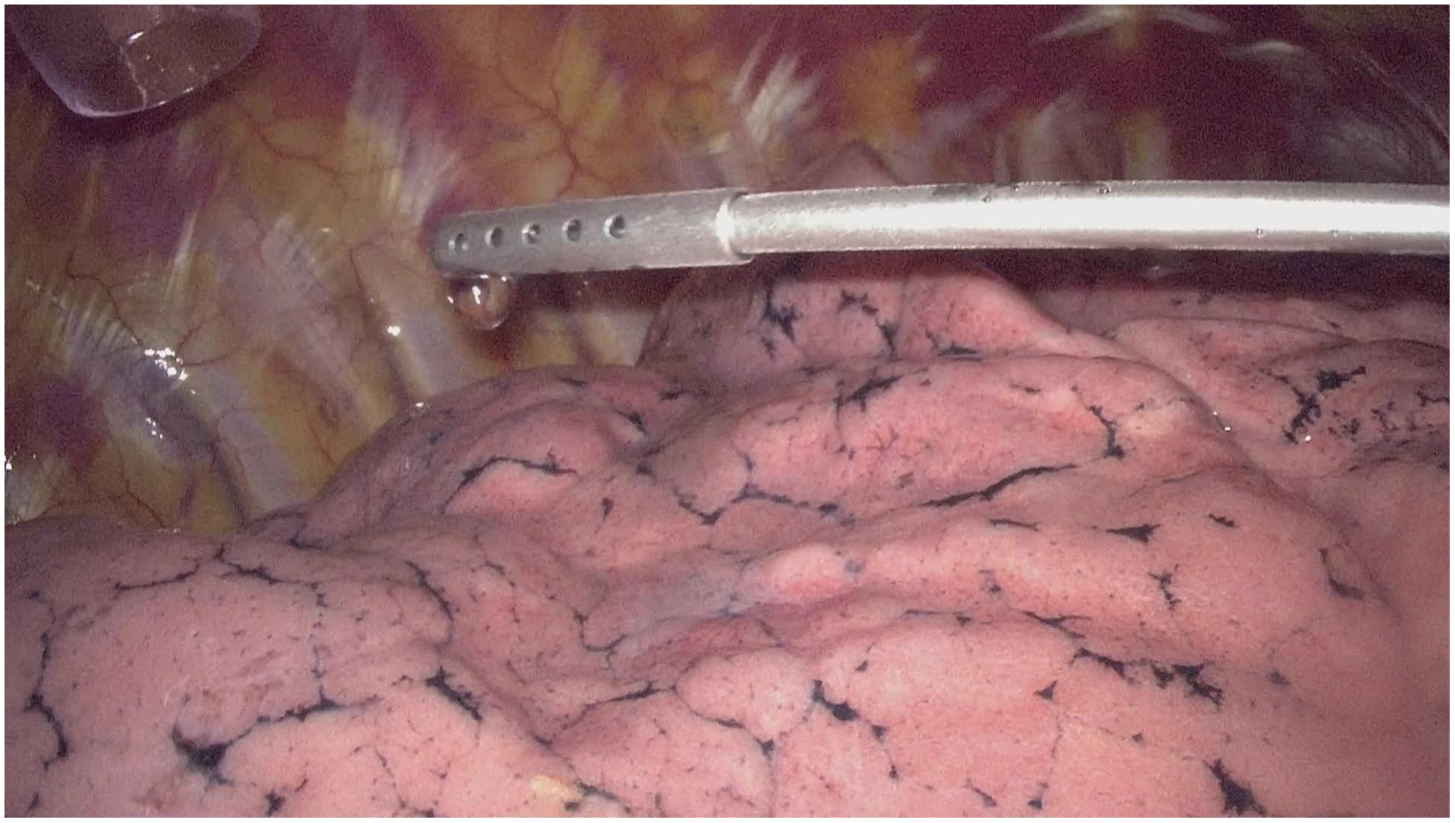
2% Lidocaine was sprayed on the lung surface and visceral pleura.

**Figure 3:**
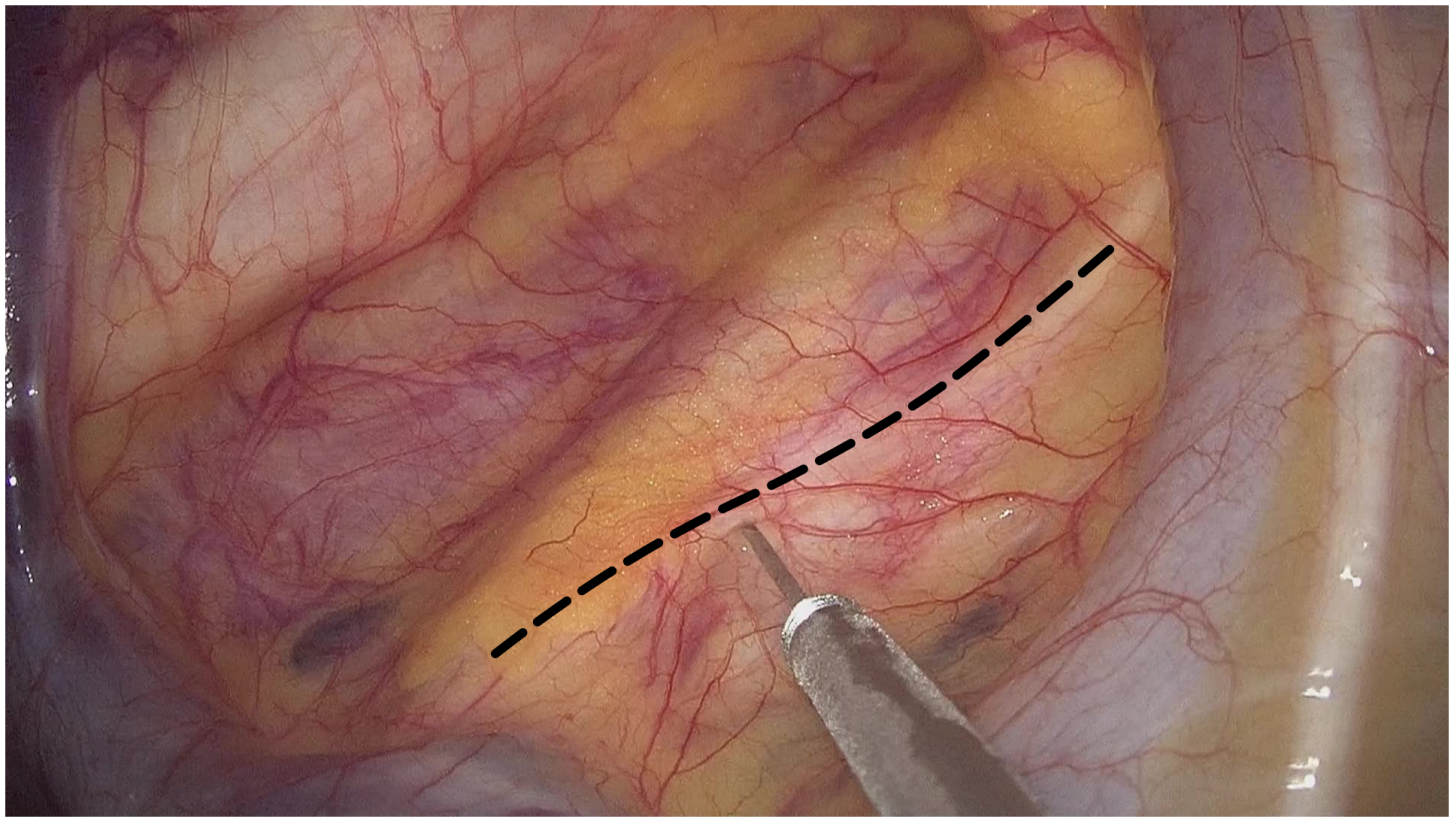
2% lidocaine was injected into the right intrathoracic vagal nerve trunk (black dotted line) at the beginning of thoracic phrase in the operation.

The non-intubated anesthesia with discontinuous spontaneous ventilation was accomplished by laryngeal mask (**Figure 1**). During the thoracic phrase, as shown in **Figure 1**, manipulation holes for the operator were placed in the seventh intercostal space at the right middle axillary line (10mm) and the fourth intercostal space at right posterior axillary line (5mm). The manipulation hole for the assistant was placed in the fourth intercostal space at right anterior axillary line by enlarging the trocar incision (4cm). The observational hole was placed in the sixth intercostal space at the right middle axillary line (10mm). 2% Lidocaine was sprayed on the lung surface and visceral pleura (**Figure 2**) and intrathoracic vagal nerve blockade was performed by injection of 2% lidocaine into the right intrathoracic vagal nerve trunk at the beginning of surgery (**Figure 3**). During the thoracic phase, on the basis of keeping oxygen saturation above 90%, spontaneous respiration frequency was controlled to 10-15 times/min and the tidal volume 200-300 mL in order to reduce the mediastinal oscillation and produce a greater surgical field. In addition, the integrity of contralateral mediastinal pleura was ensured during the whole process of esophagus dissociation for preventing contralateral pneumothorax. Abdominal surgery was successfully completed with CO_2_ pneumoperitoneum pressure controlled < 13 mmHg and mechanical anastomosis between tubular stomach and cervical esophagus was achieved in all these patients.

In our cancer center, the adjustment of pulmonary ventilation parameters during the operation mainly follows the principle: to maintain a relative shallow and fast breathing is conducive to the manipulation of the surgeon. If tidal volume of the patient is too large, which affects the manipulation of surgeon, a small dose of cisatracurium (1mg) will be administered intravenously to reduce the tidal volume, and manual assisted ventilation will be used if necessary. The reference for adjusting pulmonary ventilation parameters is to keep carbon dioxide partial pressure (PaCO_2_) less than 80mmHg. Tracheal intubation with a single-lumen endotracheal tube was always prepared for emergency situations during all the surgical procedure, however, all surgical processes were accomplished without conversion to endotracheal intubation.

In our hospital, preoperative preparation involves educating patients on mastering the correct and effective coughing techniques in order to ensure the efficient clearance of respiratory secretions postoperatively. The postoperative recuperation measures encompass respiratory tract management (sputum aspiration by fibrobronchoscopy if necessary) and individually tailored early enteral and parenteral nutritional support, which is primarily overseen by the Department of Nutrition.

The characteristics and perioperative parameters of the four patients were show in **Table 1**. All these patients received R0 esophagectomy and developed no distinct postoperative complications (Clavien-Dindo grade III-IV (7)) including anastomosis leakage and hoarseness. Without evidence of anastomosis leakage in barium meal examination 7-8 days after surgery, oral intake was resumed in all patients and they were discharged 11 to 14 days after surgery (**Table 1**). The number of resected lymph nodes in mediastinum were 21-27, while the number of resected lymph nodes in total were 31-37. The highest and lowest intraoperative oxygen saturation and the peak end-tidal carbon dioxide (EtCO_2_) and PaCO_2_ were reported in **Table 1**, this result revealed that safety of the NIVATS depended on pulmonary ventilation (PaCO_2_) but rather than oxygen saturation.

**Table 1:** Characteristic and perioperative parameters of the four patients.

Variable	Case 1	Case 2	Case 3	Case 4
Gender	Male	Male	Female	Female
Age (years)	75	67	68	55
BMI (kg/m^2^)	18.59	19.49	20.92	21.45
Concomitant disease	No	No	No	No
The date of surgery	24 Nov 2022	25 Nov 2022	1 Dec 2022	10 Apr 2023
Operation duration (min)	320	390	275	185
Blood loss volume (mL)	50	60	35	25
Peak EtCO_2_/PaCO_2_ during operation (mmHg)	51/59	60/63	55/59	51/58
highest SpO_2_/SaO_2_ during operation (%)	100/100	100/100	100/100	100/100
Lowest SpO_2_/SaO_2_ during operation (%)	95/96	94/96	97/97	94/96
Lymphocyte level (POD 1,×10^9^/L)	2.23	0.75	1.08	1.82
Lymphocyte level (POD 2,×10^9^/L)	0.27	0.77	0.73	0.39
Lymphocyte level (POD 3,×10^9^/L)	0.59	0.85	0.45	0.46
The throat discomfort	No	No	No	No
Barium meal examination time (POD)	7	7	8	7
Postoperative hospital stay (days)	11	12	11	14
Resected lymph nodes in mediastinum	21	25	27	26
Resected lymph nodes in total	31	34	37	42
Pathological subtype	ESCC	ESCC	ESCC	ESCC
Pathological stage	T3N1M0	T3N2M0	T1bN0M0	T1bN2M0
Adjuvant therapy	No	No	No	RC
Death	No	No	No	No
Recurrence	No	No	No	No

BMI, body mass index; EtCO_2_, end-tidal carbon dioxide; PaCO_2_, carbon dioxide partial pressure; POD, postoperative day; RC, radiochemotherapy; SaO_2_, arterial oxygen saturation; SPO_2_, pulse oximetry-derived oxygen saturation.

With regard to the adjuvant therapy, Case 4 received concurrent radiochemotherapy 2 months postoperatively. According to the schedule, these patients are recommended for follow up in the outpatient clinic every 3 months for the first 2 years after the surgery, every 6 months for the next 3 years, and annually thereafter. Until the date of data collection (10 October 2023), there has no neoplasm been detected by computed tomography (CT) in the thoracic cavity and upper abdomen, and enlarged supraclavicular lymph nodes have not been detected in the neck. There was a minimal amount of pleural effusion discovered in one patient’s CT scan (Case 2), however, as the patient did not exhibit any symptoms such as dyspnea or other related discomfort, no immediate corrective measures were deemed necessary.

## 3 Discussion

Non-intubated video-assisted thoracic surgery (NIVATS) has been successfully performed in lobectomy and segmentectomy in recent years (8). Moreover, NIVATS is favor to conventional intubation surgery for less invasiveness and shorter recovery time postoperatively (9), but the safety and efficacy of NIVATS in esophagectomy for esophageal cancers remains unclear. Exploration of this report contributed to provide a deep insight into the practice of NIVATS with discontinuous spontaneous ventilation in patients with esophageal cancers. Theoretically, this approach with non-invasive ventilation by laryngeal mask-assisted anesthesia may benefit from diminished residual effects of muscle relaxants on systemic autonomic nerves and facilitating postoperative recovery.

None of the four patients converted to endotracheal intubation with one lung mechanical ventilation in this study, albeit with conversion rate of 1-2% reported by Zhihua Guo and colleagues (10). Although the peak EtCO_2_/PaCO_2_ during operation was 51-63 mmHg in these four cases, this transitory hypercapnia resolved by increasing ventilation manually was permissive, given that moderate hypercapnia with EtCO_2_ 50-60 mmHg is helpful to improve lung oxygenation without compromise with hemodynamics and surgical maneuvers (11). Laryngeal mask has been proved to be safe in esophagectomy, but the successful airway management require the careful observation of anesthetist to prevent aspiration and air leakage (12). So there is no doubt that an experienced team of surgeons, anesthetists and nurses is the key point of a successful NIVATS (13). There is no consensus about the indication for the standard tracheal intubation in esophagectomy with NIVATS. In addition, NIVATS was performed in selected patients in our cancer center, we haven’t encountered any situation that requires conversion to tracheal intubation during NIVATS so far. Therefore, the indications for conversion to tracheal intubation mentioned below are based on our surgical experience but not the surgical accidence we have faced during the operation. The potential indications for conversion to tracheal intubation are: 1) esophagus cannot be effectively mobilized due to the violent mediastinal oscillation during thoracic phrase; 2) persistent hyoxemia or carbon dioxide retention due to the limited pulmonary ventilation is observed.

The number of retrieved lymph nodes in mediastinum was 21-27, suggesting that thorough lymphadenectomy was capable in minimally invasive esophagectomy under NIVATS, as described in lobectomy and segmentectomy by Jun Liu et al. (7). We carefully selected these four patients with BMI < 25kg/m^2^ given that obesity patients with BMI > 30kg/m^2^ have the anatomical disadvantage of smaller thoracic cavity causing by higher mediastinum-to-chest ratio and a higher position of the diaphragm (14). The extent of lymph node resection in esophagectomy under NIVATS for obesity patients remains to be further explored.

Intriguingly, it is reported that NIVATS with spontaneous ventilation is associated with a higher proportion of natural-killer cells and total lymphocyte count postoperatively than those with endotracheal intubation anesthesia and mechanical one lung ventilation (15). One possible explanation is that mechanical one lung ventilation increases the alveolar concentrations of proinflammatory mediators, e.g. interleukin-6 and tumor necrosis factor-α, which may spillover into systemic circulation and play a vital role on the postoperative lymphocyte response and natural-killer activity (16). At the date of data collection in this report, there is no recurrence detected in these four patients, but whether or NIVATS with spontaneous ventilation has an impact on the immune cells and the clinical outcomes in esophagectomy is an interesting investigation in the future.

Given that the utilization of robotic assisted esophagectomy offers distinct advantages, including higher lymph nodes harvest number but rather than length of hospital stay and other perioperative outcomes (17, 18). As such, the potential synergy between NIVATS and robotic esophagectomy in further accelerating recovery warrants exploration.

The minimally invasive esophagectomy with NIVATS may avoid the potential complications that intubation may result in, and may be helpful to judge the reason of postoperative hoarseness. On the other hand, patients may benefit from the reduced residual effects of muscle relaxants which may contribute to promote the postoperatively effective and self-directed expectoration and then reduce the pulmonary complications. This approach was alignment with the core idea of enhanced recovery pathways. According to our limited data, the short-term oncology outcomes were comparable to the standard techniques with tracheal intubation, but there is no doubt that the safety and feasibility of this NIVATS is warranted to be determined.

Our study has several limitations. Firstly, it is a retrospective report with a relative small sample size. Therefore, our results need further validation in larger samples with direct comparison to standard tracheal intubation approach. All included patients did not receive lung function test postoperatively which prevent us from analyzing the characteristic of NIVATS in protecting lung function. Moreover, the short follow-up duration precludes recurrence and survival analysis of this study. But the major objective of this report was to explore the primary feasibility of this approach, the recurrent data and survival analysis will be reported in the future.

## 4 Conclusions

Minimally invasive esophagectomy with non-invasive ventilation by laryngeal mask-assisted anesthesia is probably a promising alternative approach for selected patients with ESCC. The exploration of safety and effectiveness for this approach in a larger scale study is warranted.

## Data availability statement

The original contributions presented in the study are included in the article/supplementary material. Further inquiries can be directed to the corresponding authors.

## Ethics statement

The studies involving humans were approved by the Ethics Committee of Gaozhou People’s Hospital (GYLLPJ-2023092). The studies were conducted in accordance with the local legislation and institutional requirements. The participants provided their written informed consent to participate in this study. Written informed consent was obtained from the individual(s) for the publication of any potentially identifiable images or data included in this article.

## Author contributions

WC: Writing – original draft, Investigation, Data curation, Conceptualization. JZ: Writing – original draft, Methodology, Investigation, Funding acquisition, Formal analysis, Data curation. JH: Writing – original draft, Software, Methodology, Formal analysis, Data curation. HC: Writing – original draft, Methodology, Data curation. CF: Writing – original draft, Resources, Methodology, Data curation. YX: Writing – original draft, Resources, Formal analysis. HH: Writing – original draft. YC: Writing – original draft, Resources, Formal analysis. CL: Writing – original draft, Resources, Methodology. BW: Writing – original draft, Resources. WD: Writing – review & editing. WL: Writing – review & editing.
